# A Time-Domain Analog Spatial Compressed Sensing Encoder for Multi-Channel Neural Recording

**DOI:** 10.3390/s18010184

**Published:** 2018-01-11

**Authors:** Takayuki Okazawa, Ippei Akita

**Affiliations:** Department of Electrical and Electronic Information Engineering, Toyohashi University of Technology, 1-1 Hibarigaoka, Tempaku-cho, Toyohashi, Aichi 441-8580, Japan; takayuki.okazawa.jp@ieee.org

**Keywords:** compressed sensing, time domain analog, spatial

## Abstract

A time-domain analog spatial compressed sensing encoder for neural recording applications is proposed. Owing to the advantage of MEMS technologies, the number of channels on a silicon neural probe array has doubled in 7.4 years, and therefore, a greater number of recording channels and higher density of front-end circuitry is required. Since neural signals such as action potential (AP) have wider signal bandwidth than that of an image sensor, a data compression technique is essentially required for arrayed neural recording systems. In this paper, compressed sensing (CS) is employed for data reduction, and a novel time-domain analog CS encoder is proposed. A simpler and lower power circuit than conventional analog or digital CS encoders can be realized by using the proposed CS encoder. A prototype of the proposed encoder was fabricated in a 180 nm 1P6M CMOS process, and it achieved an active area of 0.0342 ${\mathrm{mm}}^{2}\;/\mathrm{ch}.$ and an energy efficiency of 25.0 pJ/ch.·conv.

## 1. Introduction

Investigating the network of the brain is the fundamental mission of neuroscience, and neural probes play an important role in this task [1]. By applying MEMS technology for the fabrication of neural probes [2], neural probe arrays, which have multiple electrodes on a single probe [3,4], can be fabricated. Furthermore, with miniaturized neural probes, integrated neural recording microsystems with CMOS LSI have been realized [5,6,7,8,9,10,11,12,13,14,15,16,17,18,19,20,21,22,23]. The number of channels on a neural probe array becomes doubled in 7.4 years, which is similar to *Moore’s law* [24]. Therefore, a greater number of recording channels and higher density of front-end circuitry is required for exponentially increasing the number of recording channels.

Since AP has a bandwidth of 100 Hz to 10 kHz [25], a high-speed data transmission is needed. For example, a 10 bit, 20 ksps/ch., and 100 channel neural recording system requires 10 bits × 20 ksps × 100 ch. = 20 Mbps bandwidth data transmission. If the system requires 1000 simultaneously recording channels, the data bandwidth becomes 200 Mbps, which is unrealistic for implantable applications. Hence, a data compression technique is inescapably required for multiple-channel neural recording systems [25].

Compressed sensing (CS) [26,27] is a data reduction technique that can be realized by using a simple operation. In a measurement system based on Nyquist–Shannon sampling theorem, superfluous sampling is required in spite of the *sparse* information in the signal. CS is a mathematical framework that can ensure accurate reconstruction from fewer measured data, which is observed using simple matrix-vector multiplication. Since CS encoder does not require any additional circuits such as feature extractor to compress the data by other compression methods (e.g., spike detector), which causes an increase in chip area, CS-based measurement systems can be expected to reduce the chip area and power consumption [28].

Although CS encoders have been proposed previously [28,29,30,31,32,33,34,35], the hardware cost of a matrix-vector multiplier for CS encoder is not small enough for multi-channel neural recording devices, and thus, the reduction of hardware cost is an essential issue. The hardware cost depends on the CS encoder architecture, which can be classified into temporal CS [28,31,32] and spatial CS [33,34,35]. The temporal CS, especially by a digital implementation, has an advantage in terms of energy efficiency in high-resolution measurement [28]. However, for multichannel measurement using the temporal CS approach, the digital product-sum operation circuit has to be parallelized for each measurement channel, increasing chip area per measurement channel. In the spatial CS [33,34,35], on the other hand, the product-sum operation circuit can be shared with a plurality of channels. Thus, it is suitable for realizing multiple-channel measurement systems. In addition, since an operating frequency of a data converter can be reduced owing to analog CS encoding in front of the data converter, lowering the dynamic power consumption. As analog spatial CS approach for multi-channel neural recording has been proposed in Ref. [34]. It succeeded the reconstruction of 16 channel APs with 20 ksps. However, an analog product-sum circuit with large power is required for area-efficient implementation. A ΔΣ-ADC-based CS encoder has also been proposed [35]. It can eliminate the need for an additional circuit for CS encoder by realizing product-sum operation by using ΔΣ modulator in ADC. However, ΔΣ ADC essentially requires over sampling to obtain the desired accuracy, resulting in high system clock frequency for the neural recording.

In this paper, a novel low-power and area-efficient time-domain analog CS encoder is proposed. The proposed CS encoder is simpler and has a more area-efficient architecture compared to a digital CS encoder, as it uses a time-domain analog product-sum operation circuit. In addition, since the time domain analog circuit is asynchronous operation, a high clock frequency is not required for the proposed encoder. Furthermore, the product-sum operation circuit of the proposed encoder operates with a small static current, and therefore, it can realize lower power and a more area efficient CS encoder than conventional CS encoders.

The rest of the paper is organized as follows. In Section 2, a theoretical background of CS is introduced. In Section 3, the concept of the proposed CS encoder is presented. Details of the proposed CS encoder system and its design methodology are described in Section 4. In Section 5, the measurement results of the fabricated proposed CS encoder prototype are discussed. Finally, the paper is concluded in Section 6.

## 2. Theoretical Background of CS

**Figure 1:**
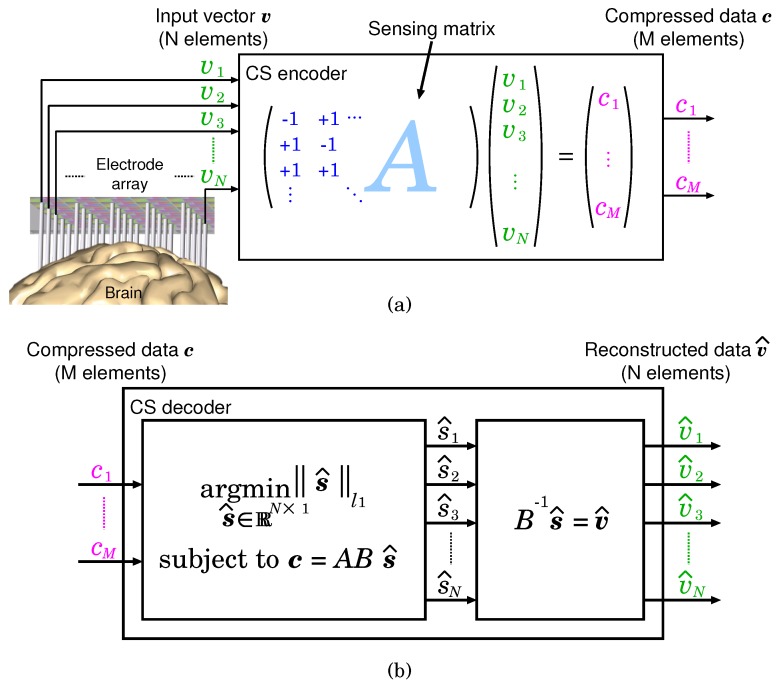
(**a**) Signal measurement (encode) and (**b**) reconstruction (decode) process based on CS.

In this section, a theoretical background of signal compression and reconstruction based on CS is introduced. CS is a mathematical framework that ensures accurate data reconstruction from fewer measured data than that is required for the conventional Nyquist–Shannon-based signal acquisition, and has been established by Donoho [26], Candes [27], and Tao [27]. Figure 1a shows the neural signal measurement (encode) process based on CS. In CS theory, an input signal vector $v\in R^{N\times 1}$ can be represented as

v=Bs,

where $B\in R^{N\times N}$ is a basis for representing ***v***, and $s\in R^{N\times 1}$ is coefficient vector [36]. When the number of non-zero elements of ***s*** is K≪N, vector ***v*** is *K*-sparse on the basis *B*. If ***v*** has sparsity on the arbitrary basis *B*, ***v*** can be represented by using vector $c\in R^{M\times 1}$ (M≤N), which has fewer dimensions, as

c=Av,

where $A\in R^{M\times N}$ is a sensing matrix for an incoherent sampling [37] . It is known that Bernoulli matrix in which all the entries are either +1 or −1 can be used as the sensing matrix [28]. A compression ratio (CR) can be defined as CR=N/M, and ***c*** becomes uncompressed data when N=M.

The reconstruction (decode) process is shown in Figure 1b. As effective signal reconstruction methods, $l_{p}$-norm minimization [38] and block sparse Bayesian learning (bSBL) [39] are widely known. $l_{p}$-norm minimization derives a sparse vector by minimizing $l_{p}$-norm ${∥a∥}_{p}=\sum_{n=1}^{N}{\left|a_{n}\right|}^{p}$ (0≤p<1). bSBL can improve reconstruction performance by applying Bayesian learning. In this study, the classical $l_{1}$ norm minimization [26,27] is used to reduce the amount of calculation. The input vector ***v*** can be reconstructed by solving the convex optimization problem about $l_{1}$-norm as

$$\underset{\hat{s}\in R^{N\times 1}}{\mathrm{argmin}}{∥\hat{s}∥}_{1}\;\;subject\;to\;\;c=AB\;\hat{s},$$

where $l_{1}$ norm of vector $a\in R^{N\times 1}$ is defined as ${∥a∥}_{1}=\sum_{n=1}^{N}\left|a_{n}\right|$ [28]. Since the basis *B* is not required for the signal encode process, the CS encoder does not require any feature extraction for signal compression. Therefore, the CS-based measurement system can reduce the system complexity.

## 3. Time Domain Analog Signal Processing for CS Encoder

**Figure 2:**
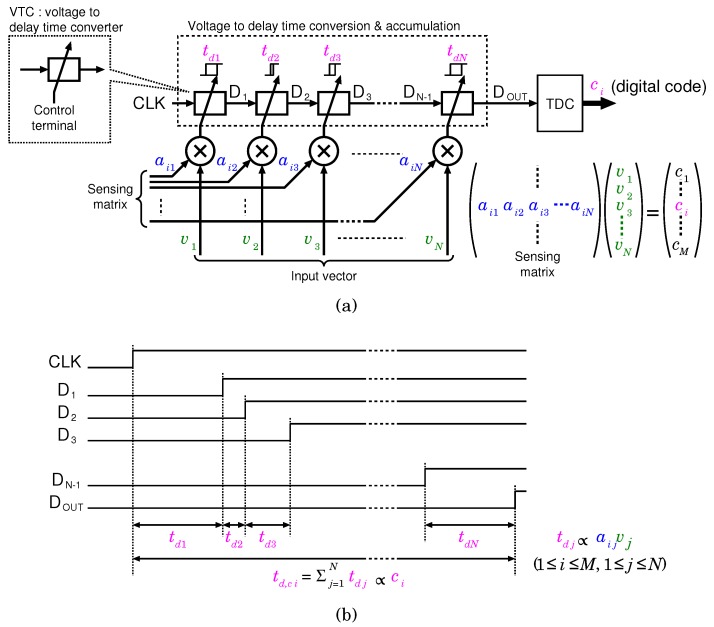
(**a**) Concept of the proposed time-domain analog spatial CS encoder and (**b**) timing chart for expressing its operation.

Figure 2a shows the concept of the proposed time-domain analog spatial CS encoder. The CS encoder executes the following product-sum operation per a clock cycle between an input vector ***v*** and a row of matrix *A* as

$$c_{i}=\sum_{j=1}^{N}a_{ij}v_{j},$$

where $c_{i}$ is an element of result vector ***c***, $a_{ij}$ is an element of the matrix *A*, $v_{j}$ is an element of ***v***, and 1≤i≤M is the number of elements in the vector ***c***. In spatial CS architecture, frame rate should be larger than twice of signal bandwidth, because CS is applied only for spatial domain and each frame is encoded. Therefore, if a input signal bandwidth including AP is 10 kHz [25], a frame rate corresponding to sampling frequency, $f_{s}$, should be greater than 20 kHz to guarantee the Nyquist–Shannon sampling theorem. The product-sum operation is executed by using cascaded voltage-to-delay-time converters (VTCs), which convert the control voltage into a time-domain signal. A product, $a_{ij}\times v_{j}$, is set to each control terminal of the VTCs, and a conversion cycle starts at the rising edge of CLK. The timing diagram of the encoder is shown in Figure 2b. When CLK rises, the first VTC starts a voltage-to-delay-time conversion. After a delay of $t_{d1}$, which corresponds to the product $a_{ij}\times v_{j}$, the first VTC raises its output $D_{1}$. Then $D_{1}$ rises, and the second VTC starts the conversion in the same manner. Thus, the delay time generated by the VTCs are accumulated, and the total delay time, $t_{d,ci}$, corresponding to $c_{i}$ appears between CLK and $D_{\mathrm{OUT}}$. Finally, $t_{d,ci}$ is converted into a digital code $c_{i}$ by the time-to-digital converter (TDC).

**Figure 3:**
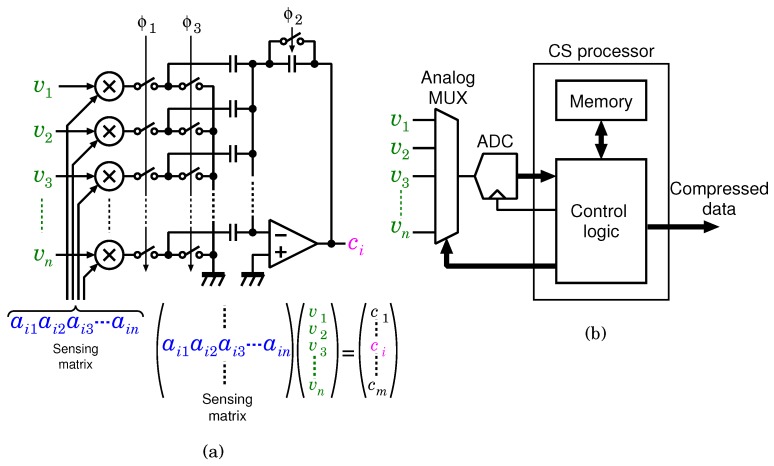
Conventional CS encoder implementation of (**a**) voltage-domain analog circuits, and (**b**) digital circuits.

Conventional analog CS encoders, including the voltage domain [33] and digital implementation shown in Figure 3, tend to be power hungry because of the following reason: In the analog implementation, the number of samples is reduced in front of the ADC by using a product-sum circuit composed of a voltage-domain mixer and a switched-capacitor adder. Similar to the time-domain implementation, the analog CS encoder can execute the product-sum operation per a clock cycle. However, the switched-capacitor adder requires an operational amplifier, which satisfies the fast settling condition, resulting in increased power consumption. The digital implementation shown in Figure 3b requires full-sampled data before compression. It is similar to the other digital signal compressors which require large-scale memory to store the data before compression. When the product-sum circuit is realized by using a single accumulator to reduce hardware cost, a system clock frequency of $M\times N\times f_{s}$ is required for the 1-frame encode. For example, if $f_{s}=20\;\mathrm{kHz},M=N=20$ (uncompressed), and the required system clock frequency is 8MHz. Since the dynamic power consumption of the clock synchronization circuit is proportional to the system clock frequency, a higher system clock frequency is undesirable. On the other hand, the proposed CS encoder is an analog circuit, which can be mainly composed of logic elements, and therefore, the proposed encoder can essentially reduce its power consumption. Moreover, since the operation of the proposed encoder is based only on delay propagation, the total number of transition cycles in the proposed encoder can be lowered than that in the conventional digital implementation. Therefore, the power consumption of the proposed CS encoder can be significantly reduced.

## 4. Design of the Proposed CS Encoder

### 4.1. Overview of the Entire Operation

**Figure 4:**
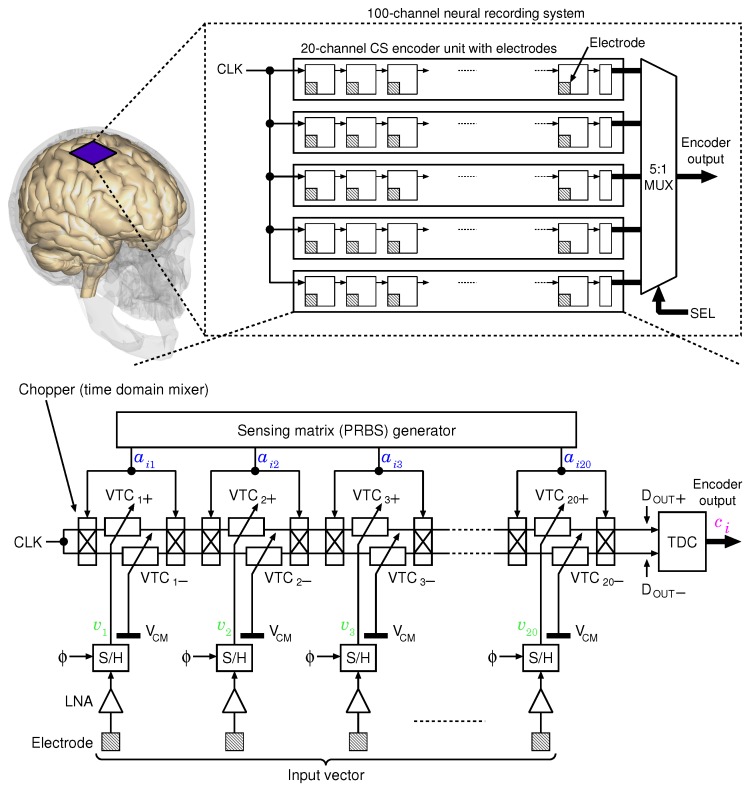
Block diagram of the proposed 100-channel time domain analog spatial CS encoder system.

Figure 4 shows the block diagram of the proposed CS encoder. It comprises 5 measurement units with 20 electrodes, and consequently, the system can simultaneously measure 100 channels of a neural signal. In this design, each input signal of the channel is represented as a pseudo-differential signal, which is converted by ${\mathrm{VTC}}_{j+}$ and ${\mathrm{VTC}}_{j-}$ (1≤j≤20), and the control voltages of ${\mathrm{VTC}}_{j+}$ and ${\mathrm{VTC}}_{j-}$ are set as input voltage $v_{j}$ and reference voltage $V_{CM}$ for $v_{j}$, respectively. The control voltages are kept constant by the sample and hold (S/H) circuits during 1-frame conversion. The S/H circuits sample their inputs ($V_{CM}$ or $v_{j}$) when the sampling clock ϕ is high. Multiplication with ±1 is executed by using choppers as a time-domain multiplier. The resulting encoder output is represented by the time difference between the rising edges of $D_{\mathrm{OUT}+}$ and $D_{\mathrm{OUT}-}$, and is converted into a digital code by the TDC.

**Figure 5:**
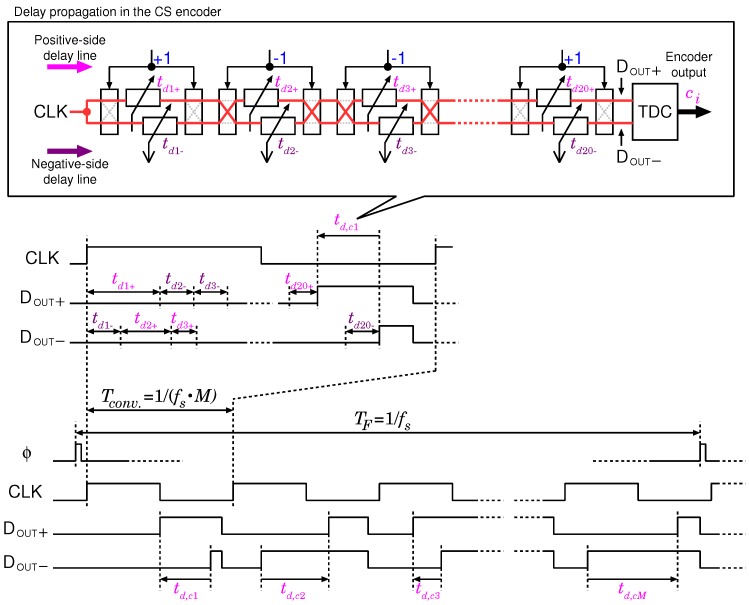
Timing diagram of the proposed CS encoder.

The timing diagram of the CS encoder is shown in Figure 5. This encoder executes a product-sum operation between a row of the sensing matrix and the input vector per a single clock cycle. ${\mathrm{VTC}}_{j+}$ and ${\mathrm{VTC}}_{j-}$ of each channel are assigned to a positive or negative delay line, respectively, by the chopper. Delay accumulation of the positive and negative delay lines are asynchronously executed, and the product-sum output appears in the relationship between the delay time $t_{d}$ and the control voltage $V_{CTL}$ of the VTC, which can be represented by a linear function as

$$t_{d}\left(V_{CTL}\right)=\alpha \times V_{CTL}+\beta ,$$

where α and β are constants. When the delay times of the ${\mathrm{VTC}}_{j+}$ and ${\mathrm{VTC}}_{j-}$ are defined as

$$t_{dj+}\equiv t_{d}\left(v_{j}\right)=\alpha v_{j}+\beta \;\;\;\mathrm{and}\;\;\;t_{dj-}\equiv t_{d}\left(V_{CM}\right)=\alpha V_{CM}+\beta ,$$

respectively, the time difference between the rising edges of $D_{\mathrm{OUT}+}$ and $D_{\mathrm{OUT}-}$ is directly proportional to the product-sum output as

$$t_{d,c_{i}}\left(v\right)=\sum_{j=1}^{N}a_{ij}\left(t_{dj+}-t_{dj-}\right)=\alpha \left\{\sum_{j=1}^{N}a_{ij}\left(v_{j}-V_{CM}\right)\right\}.$$

The upper limit of each VTC’s delay is determined by the stage number of the VTCs *N* and the frame rate $f_{s}$. Since the period of the frame is $T_{s}=1/f_{s}$, M-times product-sum operation must be executed in $T_{s}$. Therefore, the upper limit of the time for the product-sum operation is

(1) $$T_{conv.,max}=\frac{1}{f_{s}\times M}.$$

Since $T_{conv.,max}$ is the total delay time of the VTCs, the upper limit of each VTC’s delay can be expressed by using Equation (1) as

$$t_{d,max}=\frac{T_{conv.,max}}{N}=\frac{1}{M\times N\times f_{s}}.$$

When the design parameter values are set as $f_{s}=20\;\mathrm{ksps}$ and M=N=20 (uncompressed), the upper limits are determined as $T_{conv.,max}=2.5\;\mu s$ and $t_{d,max}=125\;\mathrm{ns}$.

**Figure 6:**
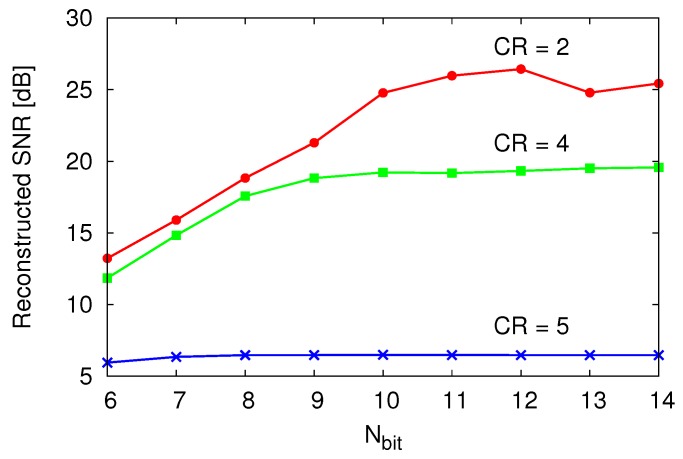
Reconstructed SNR vs. bit resolution of the TDC.

The required number of bits for TDC $N_{bit}$ must be determined so as not to degrade the reconstructed data. To determine $N_{bit}$, a system level simulation by using MATLAB was performed as shown in Figure 6, which plots the reconstructed SNR vs. the $N_{bit}$ on each compression ratio CR (=N/M). The reconstructed SNR is defined as

(2) $$SNR=-10\log\frac{\sum_{i=1}^{N}{\left(v_{i}-{\hat{v}}_{i}\right)}^{2}}{\sum_{i=1}^{N}v_{i}^{2}},$$

where $v_{i}$ is the original data, and ${\hat{v}}_{i}$ is the reconstructed data [28]. Note that noise-less TDC and VTC are assumed in the simulation. A reconstructed SNR saturation on the higher $N_{bit}$ is caused by CR, and thus, dropping the reconstructed SNR on a lower $N_{bit}$ denotes the insufficiency of $N_{bit}$. According to the result, $N_{bit}$ was decided as 10 bit in this design. The time resolution of the TDC $T_{LSB}$ can also be derived from Equation (1) and $N_{bit}$ as

(3) $$T_{LSB}=\frac{T_{conv.,max}}{2^{N_{bit}}}=\frac{1}{f_{s}\times M\times 2^{N_{bit}}}.$$

In this design, $T_{LSB}$ was decided as 1.63 ns. Note that its input-referred noise is 4.86 $\mu \;V_{\mathrm{RMS}}$, and is sufficiently lower than amplitude of AP (50 to 500 $\mu \;V_{\mathrm{PP}}$ [25]). In time-domain analog circuits, jitter corresponds to the noise in voltage-domain analog circuits, and it is defined as a timing deviation from the true operation timing. The total value of jitter is mainly determined by thermal noise represented by voltage or current source [40,41]. Since thermal noise follows Gaussian distribution, jitter value also follows Gaussian distribution. Therefore, jitter value can be discussed in statistics.

**Figure 7:**
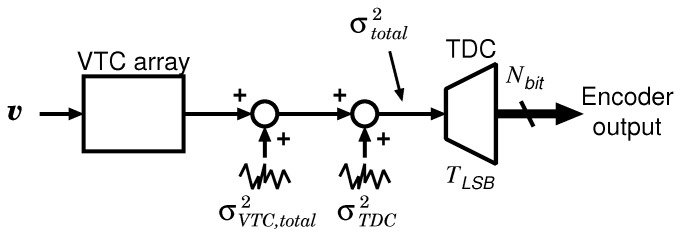
Jitter model of the proposed CS encoder.

Figure 7 shows the jitter model of the proposed CS encoder. The overall jitter $\sigma_{total}^{2}$ comprises of the output-referred jitter of VTC array $\sigma_{VTC,total}^{2}$ and the TDC input-referred jitter $\sigma_{TDC}^{2}$, where $\sigma_{VTC,total}^{2}$ is the total accumulated jitter of each VTC in the VTC array, and $\sigma_{TDC}^{2}$ is determined by the variations in the operation timings of the flip-flops and a ring oscillator in the TDC. Note that $\sigma_{TDC}^{2}$ corresponds to a sampling noise of ADC. Assuming that all the jitter deviations follow the Gaussian distribution, the overall jitter can be derived by the sum of squares as

$$\sigma_{total}^{2}=\sigma_{VTC,total}^{2}+\sigma_{TDC}^{2}.$$

In this design, the condition for satisfying the required accuracy of the CS encoder is defined as

(4) $$3\sigma_{total}=3\sqrt{\sigma_{VTC,total}^{2}+\sigma_{TDC}^{2}}\leq T_{LSB}$$

at least. As mentioned below, $\sigma_{TDC}^{2}$ is sufficiently smaller than $\sigma_{VTC,total}^{2}$ in this design. Thus, $\sigma_{TDC}^{2}$ is considered to be constant. The following subsections describe the architecture and design consideration of TDC and VTC.

### 4.2. TDC

**Figure 8:**
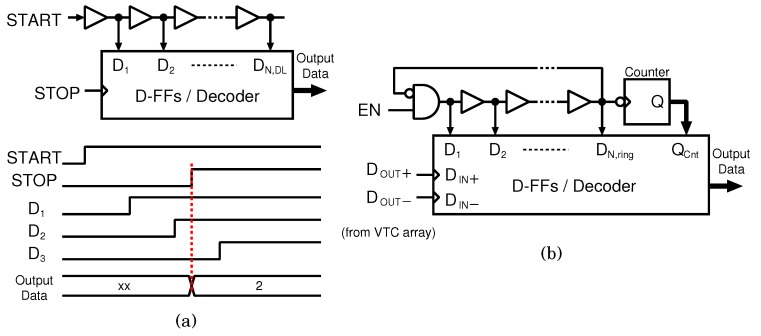
Block diagram of (**a**) delay-line-based TDC, and (**b**) ring-oscillator-based TDC.

TDC can convert the time difference between two input clock edges into a digital code with high time resolution by using a delay time of logic elements. Figure 8 shows the block diagram of a delay-line-based and a ring-oscillator-based TDC. The delay-line-based TDC, which is shown in Figure 8a, is composed of a delay line, a D-FF array, and a decoder. The TDC converts the time difference between the rising edges of the START and STOP signals. When the START signal rises, the rising edge propagates through the delay line. When the STOP signal rises, the D-FF array captures the delay propagation state, and the captured state is converted into a digital code by the decoder. Although this TDC has the simplest architecture, $2^{N_{bit}}$ stage delay lines are required to realize $N_{bit}$ bit resolution. If the desired resolution is 10 bit, a 1024-stage delay line is required. On the other hand, a ring-oscillator-based TDC, which is shown in Figure 8b, is composed of a ring oscillator, a binary counter, a D-FF array, and a decoder. In the ring-oscillator type TDC, the D-FF array captures the phase of the ring oscillator for fine conversion, and the counter measures the ring oscillator output for coarse conversion. Therefore, the ring-oscillator-based TDC requires fewer delay line stages than the delay-line-based TDC. In this design, the ring-oscillator-based TDC is employed to reduce the number of delay line stages. The following paragraphs describe the TDC jitter and the number of ring oscillator stages N.

In ring-oscillator-based TDC, the delay time of the ring oscillator’s delay cell $t_{d,Ring}$ becomes the time resolution of the TDC $T_{LSB}$. The frequency of the ring oscillator $f_{o}$ can be expressed as

$$f_{o}=\frac{1}{N_{Ring}\times t_{d,Ring}},$$

where $N_{Ring}$ is the number of ring oscillator stages. The TDC jitter $\sigma_{TDC}^{2}$ can be expressed as

$$\sigma_{TDC}^{2}=\sigma_{Ring}^{2}+\sigma_{D-FF}^{2},$$

where $\sigma_{Ring}^{2}$ is the ring oscillator jitter and $\sigma_{D-FF}^{2}$ is the timing variation when the D-FF array captures the ring oscillator phase. Since $\sigma_{D-FF}^{2}$ is a sufficiently smaller constant value than $\sigma_{Ring}^{2}$, $\sigma_{TDC}^{2}\approx \sigma_{Ring}^{2}$. Also, $\sigma_{Ring}^{2}$ can be expressed as [41]

(5) $$\sigma_{Ring}^{2}=\kappa^{2}\Delta t,$$

where κ is a proportionality constant that is determined by the circuit parameters, and Δt is the measurement time. According to [41], κ is determined by the size of the transistor for the ring oscillator (*W* and *L*), the number of ring oscillator stages $N_{Ring}$, and the current noise power spectral density in which are input to ring oscillator nodes ${\bar{i}}_{n}^{2}/\Delta f$. Since the time resolution of TDC $T_{LSB}$ is already determined by Equation 3, the transistor size for the ring oscillator cannot be modified to satisfy $T_{LSB}$. In addition, ${\bar{i}}_{n}^{2}/\Delta f$ is also not changeable in this design; only $N_{Ring}$ can be modified. However, the TDC jitter value hardly changes regardless of changing $N_{Ring}$ [41]. Therefore, $\sigma_{TDC}^{2}\approx \sigma_{Ring}^{2}$ is regarded as a constant.

In this design, since TDC consumes most of the power in the entire CS encoder, its design should be optimized to achieve lower power consumption. The power consumption of TDC $P_{TDC}$ can be expressed as

**Figure 9:**
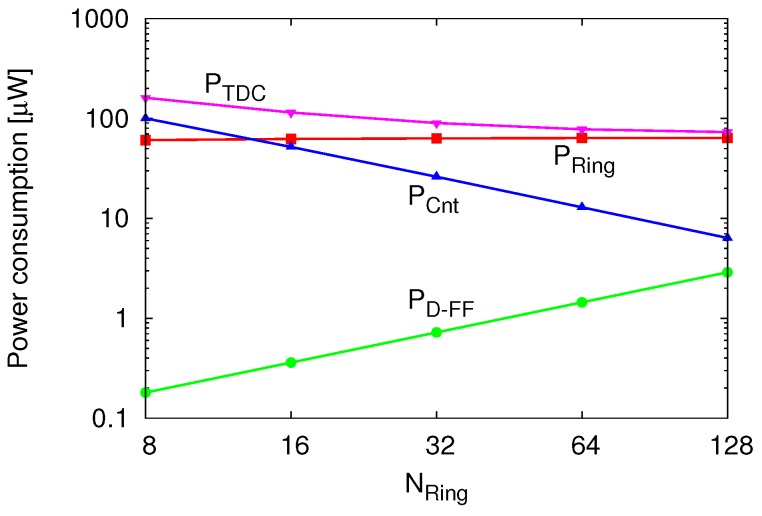
Simulation result of power consumption vs. $N_{Ring}$ during measurement.

*P_TDC_* = *P_Ring_* + *P_D-FF_* + *P_Cnt_*,

where $P_{Ring}$, *P_D-FF_* and $P_{Cnt}$ are the power consumptions of the ring oscillator, the phase capturing D-FF, and the counter for coarse conversion, respectively. Figure 9 plots the simulated power consumption vs. $N_{Ring}$ during measurement. *P_D-FF_* is sufficiently smaller than the others. $P_{Ring}$ is almost constant and occupies the majority of $P_{TDC}$. When $N_{Ring}$ is small, $P_{Cnt}$ becomes larger than $P_{Ring}$. According to the above results, increasing $N_{Ring}$ reduces the power consumption of the TDC, and it approaches the value of $P_{Ring}$. Finally, the number of ring oscillator stages is determined as N=64 in this design. To estimate the value of $\sigma_{Ring}^{2}$, 100-times transient-noise simulation is performed. According to the result, the TDC jitter with $N_{Ring}=64$ becomes sufficiently small as $3\;\sigma_{Ring}=221.6\;\mathrm{ps}$.

### 4.3. VTC

**Figure 10:**
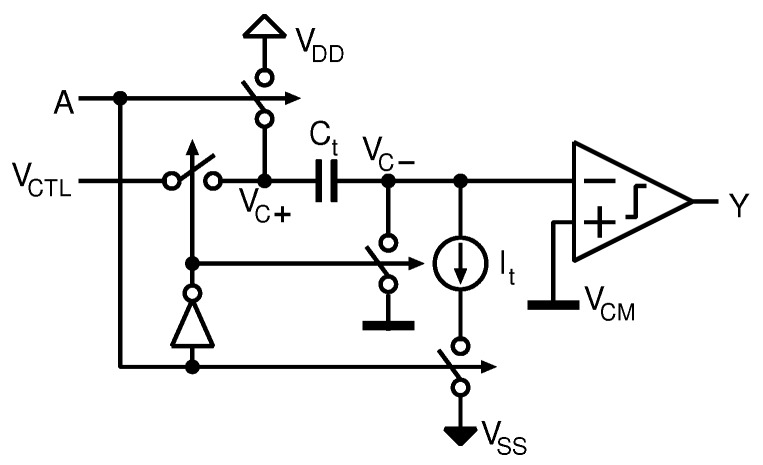
Schematic of the VTC.

**Figure 11:**
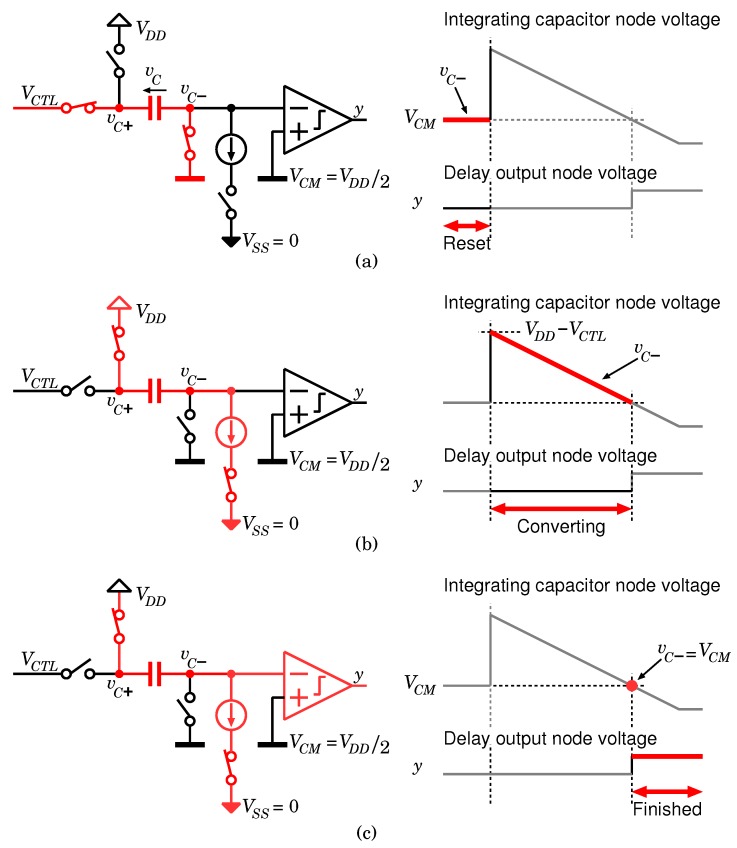
Timing diagram of VTC, (**a**) reset state, (**b**) integration state, and (**c**) conversion completed.

To realize the proposed CS encoder, a transfer function with a high linearity is required between the delay time and the control voltage. Therefore, an integrator-based architecture is composed of a capacitor and current source, as shown in Figure 10, is employed in this design. A conversion trigger signal A controls the state of VTC. When the node voltage of A is logical low, VTC is set as a reset state and the capacitor’s terminal voltage becomes $V_{CTL}-V_{CM}$ as shown in Figure 11a, where $V_{CM}=V_{DD}/2$ is a reference voltage. The voltage-to-delay-time conversion starts at the rising edge of A.

Finally, when $v_{c-}$ crosses the threshold voltage of the comparator, $V_{CM}$, the conversion is completed and the output of the comparator becomes high (Figure 11c). To ensure high linearity over a wide range of $V_{CTL}$, the delay time is controlled only by the initial voltage of the capacitor, and the integrating current and threshold voltage of the comparator are constant. Note that the comparator is composed of a simple logic inverter to reduce its operating power.

The jitter requirement for VTC can be derived by substituting $T_{LSB}$ and $\sigma_{TDC}$ into Equation 4 as $\sigma_{VTC}\leq 1.61\;\mathrm{ns}$.

**Figure 12:**
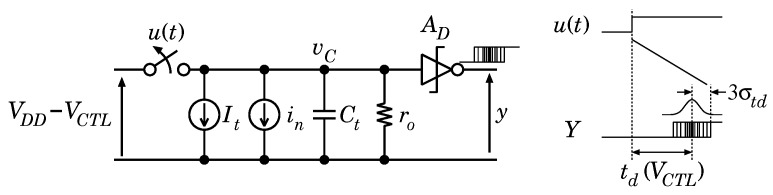
Timing diagram of VTC (**a**) reset state, (**b**) integration state and (**c**) conversion completed.

Figure 12 shows the equivalent circuit model for the integration state, where $I_{t}$ is an integrating current, $i_{n}$ is the noise current of the current source, $C_{t}$ is the integrating capacitance, and $r_{o}$ is the output resistance of the current source. The relationship between the delay time $t_{d}$ and the control voltage $V_{CTL}$ can be expressed as

(6) $$t_{d}\left(V_{CTL}\right)=\frac{C_{t}}{I_{t}}\left(\frac{1}{2}V_{DD}-V_{CTL}\right)=-\frac{C_{t}}{I_{t}}V_{CTL}+\frac{C_{t}\times V_{DD}}{2I_{t}}.$$

In this design, the measurement unit shown in Figure 4 has 20 measurement channels, and each measurement channel includes two VTCs (${\mathrm{VTC}}_{j+}$ and ${\mathrm{VTC}}_{j-}$). Thus, the total accumulated jitter for the product-sum operation $\sigma_{VTC,total}^{2}$ in Equation 4 can be represented by using a square-sum of all the VTCs’ jitter $\sigma_{td}^{2}$ as

$$\sigma_{VTC,total}^{2}=2\times 20\;\sigma_{td}^{2}.$$

Therefore, the ratio between $C_{t}$ and $I_{t}$ is derived by introducing the control voltage range and the maximum delay time into Equation 6, and $C_{t}$ can be determined as satisfying $\sigma_{VTC}\leq 1.61\;\mathrm{ns}$.

**Figure 13:**
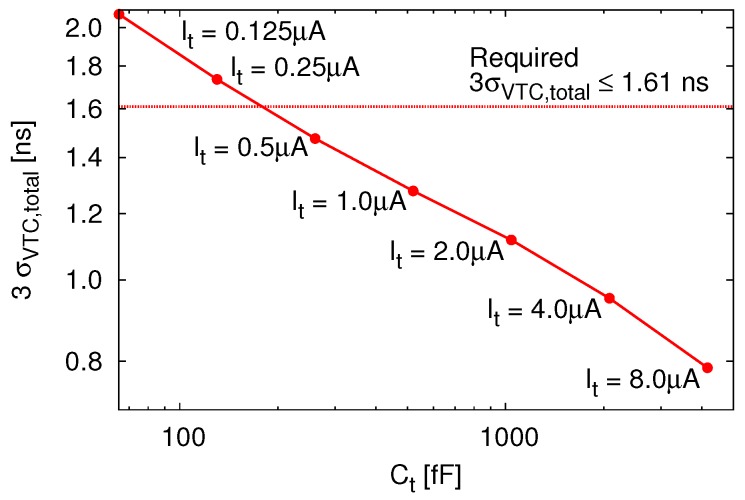
100-times transient-noise simulation result of $3\sigma_{VTC,total}$ vs. $C_{t}$.

Figure 13 plots the 100-times transient-noise simulation result of $3\;\sigma_{VTC,total}$ as a function of $C_{t}$. In this design, the capacitance of the integrator was determined as $C_{t}=521\;\mathrm{fF}$ and $I_{t}=1.0\;\mu A$ to satisfy the jitter requirement.

### 4.4. Design Constraints in Proposed Architecture

In this subsection, design constraints and trade-off of the proposed architecture are discussed. The chip area and the total number of channels are design constraints of the proposed CS encoder. These constraints provide maximum capacitance in the VTCs, and hence it decides realizable minimum jitter of the VTCs. On the other hand, if desired SNR and sampling rate are given, specifications of time resolution of the TDC and the VTC jitter are also determined. Since the specification of the VTC jitter cannot be less than the realizable one, the rest of design constraint is how balancing between the number of channels per measurement unit $N_{unit}$ and power consumption. A smaller $N_{unit}$ relaxes jitter requirement for VTC and TDC, and thus low-power implementation could be realized, while higher CR cannot be achieved because realizable maximum CR is same with $N_{unit}$. In contrast, a larger $N_{unit}$ requires low jitter for VTC and TDC, increasing power consumption. Especially, since a VTC jitter is limited by its capacitor size, the low jitter requirement for the VTC cannot be realized for much larger $N_{unit}$. Therefore, considering the above discussion, in this design, a moderate $N_{unit}$ is set as 20, and resulting maximum power consumption is 6 μW/ch. which can be expected as lower value than previous studies [33,34,35].

## 5. Measurement Results and Discussion

**Figure 14:**
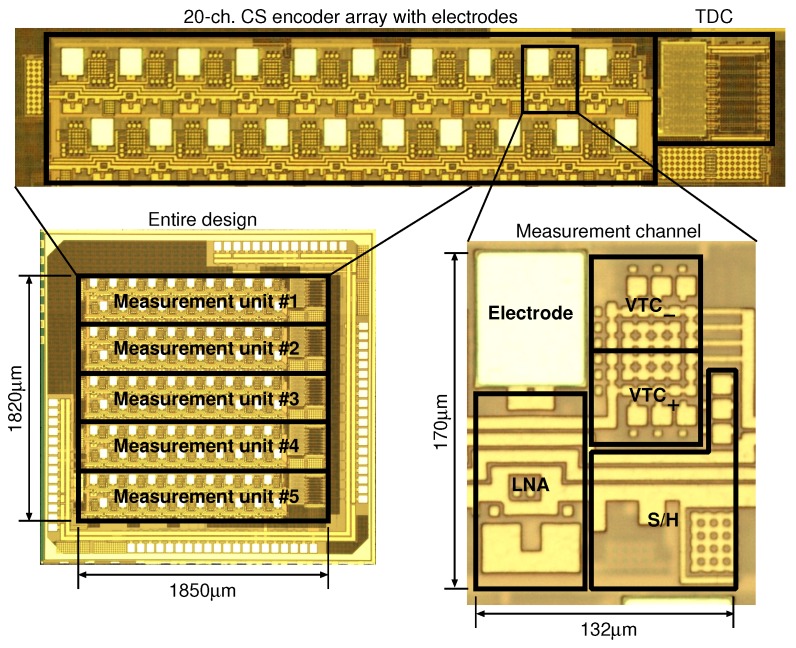
Chip microphotograph of the proposed time-domain analog CS encoder prototype.

The proposed 100-ch. time domain analog CS encoder was fabricated in a 180 nm 1P6M CMOS process, as shown in Figure 14. The active area of the prototype encoder is 1.85 mm × 1.82 mm. The frontend for each measurement channel comprises the electrode, the low-noise amplifier (LNA), the sample and hold circuit (S/H), and the two VTCs. The active area with TDC is 0.0331 ${\mathrm{mm}}^{2}\;/\mathrm{ch}.$, and without TDC is 0.0272 ${\mathrm{mm}}^{2}\;/\mathrm{ch}.$

**Figure 15:**
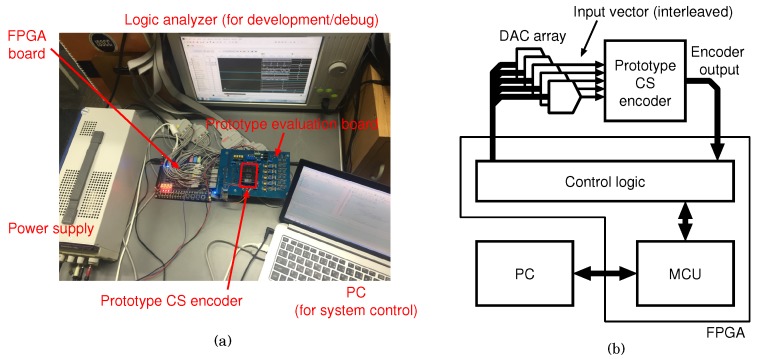
(**a**) Evaluation system for the prototype CS encoder and (**b**) its block diagram.

Figure 15 shows the evaluation environment for the proposed CS encoder. The measurement system, which is shown in Figure 15a, comprises of the prototype CS encoder, a prototype evaluation board, an FPGA board, a power supply, and a PC. A logic analyzer is used for the development and debugging of the environment. The prototype is controlled by the control logic and the micro controller (MCU), which are embedded in the FPGA. The test signal of the encoder is provided for each measurement unit as a time-interleaved voltage signal from DAC. The test signal which simulates spontaneous neuronal activity is generated on MATLAB. The details are described in Appendix A.

**Figure 16:**
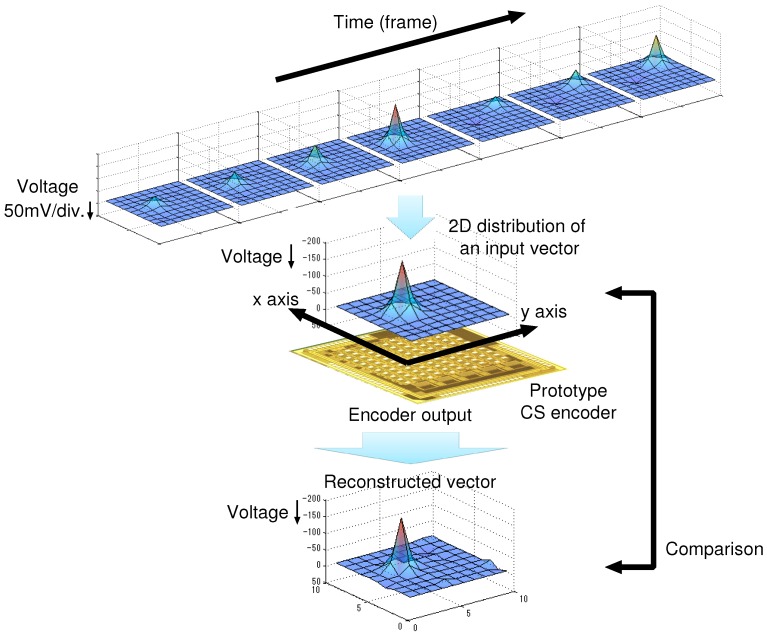
Evaluation procedure for the CS encoder.

The evaluation procedure for the CS encoder is shown in Figure 16. In this measurement, spatial test signal (input vector) were prepared for each frame and directly stored in the S/H circuits of the prototype at the beginning of the conversion frame. The test signals are generated for simulating the AP waveforms from a neural probe array, and each signal represents a 2-dimensional input voltage distribution. The acquired data from the prototype CS encoder is transferred to the PC and then reconstructed by a MATLAB-based program. Note that the reconstruction by solving the convex optimization was realized using CVX [42]. As the basis for the reconstruction, a discrete cosine transformation (DCT) matrix was selected. Data compression method based on spatial DCT has been proposed in Ref. [43] with 1/69 times data reduction at 6% root mean square error. Using DCT for data compression can imply that multi-channel APs is inherently sparse on 2D frequency domain, and thus DCT matrix can be potentially used as basis of spatial CS reconstruction.

Finally, the reconstructed signal quality is evaluated by calculating the reconstructed SNR defined in Equation (2).

**Figure 17:**
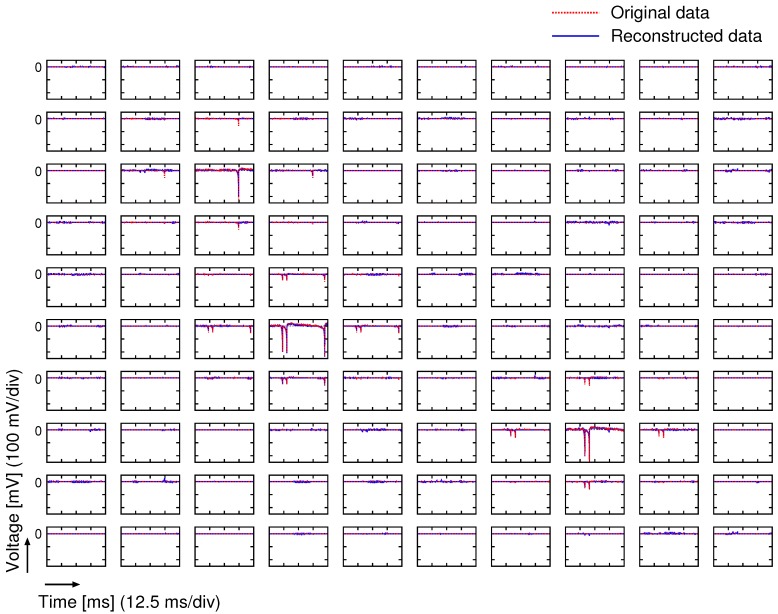
100-ch. reconstructed temporal waveforms from the compressed data encoded by the prototype CS encoder at CR = 4.

**Figure 18:**
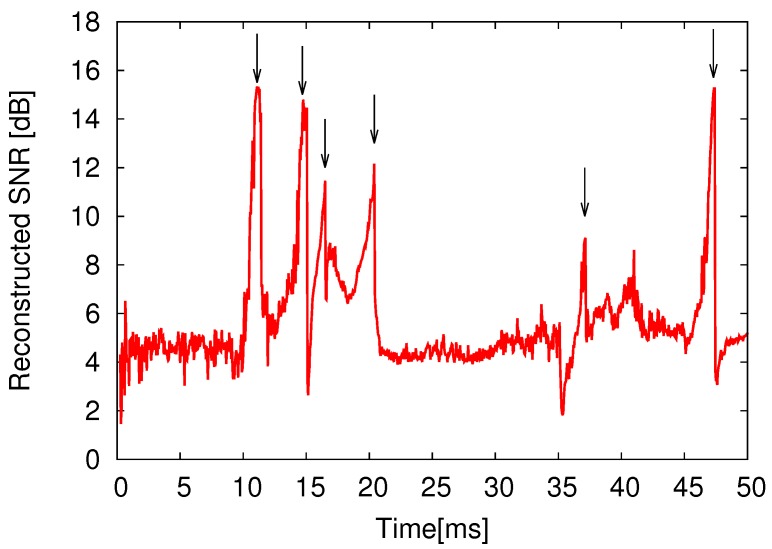
Temporal change of reconstructed SNR (CR = 4).

**Figure 19:**
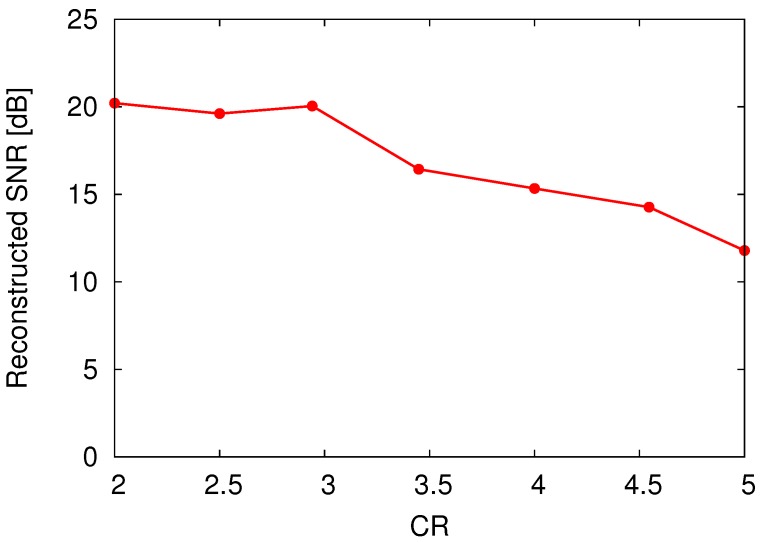
Reconstructed SNR vs. CR at *t* = 11.1 ms.

Figure 17 shows the 100-ch. reconstructed temporal waveforms from the compressed data encoded by the prototype CS encoder at CR = 4, and Figure 18 plots the temporal change in the reconstructed SNR. Note that amplitude of waveforms shown in Figure 17 indicates input voltage for VTCs. The reconstructed SNR at *t* = 11.1 ms (when the input signal becomes peak amplitude) was 15.3 dB. Since the reconstructed SNR depends on the input signal, non-sparse signal degrades the SNR. If a improved SNR is required, CR should be relaxed which means an increase in the number of sampling for each frame, *M*. In this design, the frame rate of the encoder is defined to support uncompressed condition (M=N). Therefore, the proposed encoder can control CR from 1.0 to 20 without any hardware changes. The reconstructed SNR vs. compression ratio (CR) at *t* = 11.1 ms is plotted in Figure 19. Note that the measurement results of Figure 17, Figure 18 and Figure 19 include all noises induced by the prototype chip and the measurement environment. In practical applications, influence for a spike sorting [44] must be discussed. Not to affect spike sorting performance, enough reconstructed SNR before and after spike is required for spike detection and spike classification [44]. As shown in Figure 17 and Figure 18, spike amplitude and waveform are successfully reconstructed at CR = 4, and reconstructed SNR before and after spikes (indicated with allows in Figure 18) are around 10 dB. Note that the measurement results of Figure 17 and Figure 18 include noise of the measurement environment. SNR for a spike detection and a classification requires more than 10 dB [45], recovered data do not affect spike sorting performance. The SNR was saturated at 20.0 dB with a CR lower than 3. The saturated SNR is lower than the expected MATLAB simulation result as shown in Figure 6. In addition, the SNR at CR = 4 is 15.3 dB, and it dropped over 3 dB compared to the SNR simulated using MATLAB (19.5 dB). From the simulation result shown in Figure 6, it is equivalent to a degradation of 0.7 effective number of bits (ENOB), and it is considered a systematic variation in the gain of the VTC’s, which degrades the dynamic range of the CS encoder. Since the gain variation of VTCs is not compensated in this design, it could be affected by the process, voltage, and temperature (PVT) variation. Therefore, a gain compensating technique for VTC is desired for improving the SNR. To achieve further improvement of CR and reconstructed SNR in practical in vivo measurement, an optimized basis could be used, which is obtained from uncompressed (CR = 1) recorded data by dictionary learning algorithm such as K-SVD [46]. As other solution for improving reconstructed SNR, an optimization techniques for sensing matrix have been proposed in Ref. [47]. Indeed, optimization techniques for sensing matrix can improve reconstruction performance. However, extra registers, which almost consumes 20% active area of the prototype, to contain the optimized sensing matrix is required. Thus, the technique have not been applied in this prototype, and the sensing matrix is generated on the chip by using simple linear-feedback shift register (LFSR).

**Figure 20:**
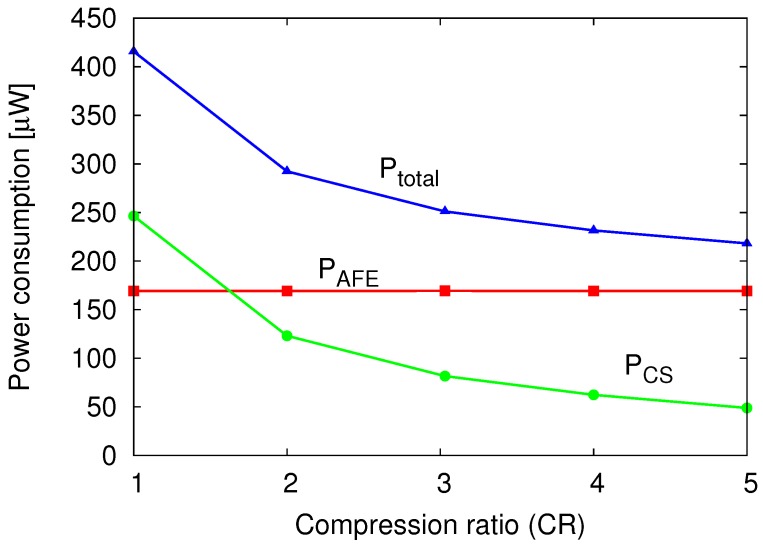
Measured power consumption vs. CR ($f_{s}=20\;\mathrm{ksps}$).

Figure 20 plots the measured power consumption vs. CR at $f_{s}=20\;\mathrm{ksps}$. The power consumption for the analog front-end (AFE) part, which comprises LNA and S/H, was constant at 1.68 μW/ch. On the other hand, the power consumption of the CS encoder parts has become inversely proportional to CR. An energy efficiency of 25.0 pJ/ch.·conv. was achieved for the CS encoder (without AFE) per channel and per conversion.

A performance comparison of the proposed CS encoder with those developed in previous works for neural recording applications is summarized in Table 1. The prototype CS encoder achieved the lowest power consumption and the smallest area compared to the encoders from previous works. Especially, the power efficiency of the CS encoder improved by about 10-times compared to the digital CS encoder.

## 6. Conclusions

In this paper, a low-power energy-efficient neural signal acquisition system, which uses the novel time-domain analog spatial CS encoder, is proposed. In this technique, the product-sum operation for the CS encoder can be executed by accumulating the delay time information. Since a major part of the proposed CS encoder can be realized by using logic elements, it can reduce power consumption and chip area compared to conventional analog or digital CS encoders. Some design parameters for the proposed encoders were considered and optimized by a trade-off between noise and power consumption.

The 100-ch. neural signal acquisition system employing the proposed time-domain CS encoder was fabricated in a 180 nm 1P6M CMOS process, and its active area is 0.0331 ${\mathrm{mm}}^{2}\;/\mathrm{ch}.$ A 100-ch. CS encode experiment was performed using the prototype CS encoder, and it achieved a reconstructed SNR of 15.3 dB and conversion energy efficiency of 25.0 pJ/ch.·conv. at $f_{s}=20\;\mathrm{ksps}$ and CR = 4. The prototype CS encoder achieved the lowest power consumption and the smallest area compared to the encoders in other previous works for neural recording applications. Therefore, the proposed time-domain spatial CS encoder is suitable for exponentially increasing multi-channel neural recording applications.

Future works are to generate a basis which is optimized for measured spatial APs and thoroughly to evaluate the performance of spike sorting with reconstructed spatial information.

## Figures and Tables

**Table 1:** Performance comparison to previous works.

Parameter	[28]	[31]	[32]	[33]	[34]	This Work
					(Simulated)	
Technology [nm]	90	180	180	180	180	180
Number of channels	1	12	16	16	16	100
Target signal type	EEG	Neural signal	LFP / AP	EEG	AP	AP
Input signal BW [kHz]	10	7	10	2	10	10
Resolution [bit]	8	12	10	10	-	10
Implementation method	Digital CS	Digital CS	Digital CS	Analog CS	Analog CS	Time-domain analog CS
Input vector type	Temporal	Temporal	Temporal	Spatial	Spatial	Spatial
Compression ratio (CR)	20	≤8	8–16	≤16	2.3	1–20
Reconstructed SNR [dB]	10 (CR = 20)	-	9.78 (CR = 8)	10.9 (CR = 4)	6.47 (CR = 2.3)	15.3 (CR = 4)
Total area [${\mathrm{mm}}^{2}\;/\mathrm{ch}.$]	0.104 (w/o LNA)	0.563	0.0489	0.0464	-	0.0331
CS encoder area (w/o AFE) [${\mathrm{mm}}^{2}\;/\mathrm{ch}.$]	0.09	-	0.0117	0.008	0.0023	0.0065
Total power efficiency [pJ/ch.·conv.]	-	-	475 (CR = 8)	238 (CR = 4)	343.5 (CR = 2.3)	92.6 (CR = 4)
CS encoder power efficiency (w/o AFE) [pJ/ch.·conv.]	-	-	241 (CR = 8)	131 (CR = 4)	53.5 (CR = 2.3)	25.0 (CR = 4)

**Figure A1:**
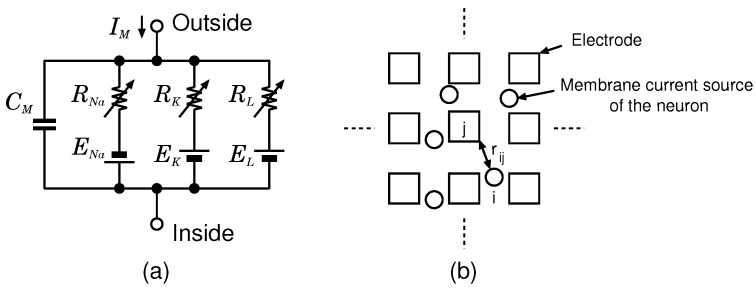
(**a**) Equivalent circuit of nerve membrane [49] and (**b**) electrodes arranged at equal intervals and randomly placed membrane current sources of neurons.

## Appendix A. Test Signal Generation

A real neural dataset which meets the conditions of our prototype chip including electrode density, electrode arrangement and the number of electrodes could not be found. Hence, in this study, the test signal which simulates spontaneous neuronal activity is generated by using MATLAB.

Upon pseudo AP signal generation, at first, membrane voltage signal of the neuron $V_{m}$ is generated by using algorithm based on Ref. [48]. Then, $V_{m}$ is converted to membrane current $I_{m}$. Finally, extracellular potential is obtained from $I_{m}$. An equivalent circuit of nerve cell’s membrane is shown in Figure A1a, where $C_{m}$ is a membrane capacity, $R_{Na}$ and $R_{K}$ are sodium and potassium resistance, respectively, $E_{Na}$ and $E_{K}$ are potassium and sodium potentials, respectively, $R_{L}$ and $E_{L}$ are leakage resistance and potential, respectively [49]. Practical parameters of the equivalent circuit shown in Figure A1a are referred to Ref. [49]. *N* neurons are randomly placed around electrodes, which are arranged at equal intervals, as shown in Figure A1b. Note that neuron density is determined by referring to Ref. [21] as 150 neurons/${\mathrm{mm}}^{2}$, averaged firing rate of neurons is 0.3 Hz, and the average number of firing neuron in the sensing area per 25 ms is 6 times. A membrane current of *i*-th (1≤i≤N) neuron can be expressed as

$$I_{m,i}=I_{i}+C_{m}\frac{\partial V_{m,i}}{\partial t},$$

where $I_{m,i}$ is an ionic current, $C_{m}$ is a membrane capacity. When $I_{m,i}$ is assumed to be a point sink current source, extracellular potential of *j*’th(1≤j≤100) electrode can be obtained as

$$\phi_{j}=-\frac{1}{4\pi \sigma }\sum_{n=1}^{N}\frac{I_{m,n}}{r_{ij}},$$

where σ is constant conductivity, and $r_{ij}$ is distance between current source and electrode [50].
